# Case report: Autoimmune glial fibrillary acidic protein astrocytopathy misdiagnosed as tuberculous meningitis

**DOI:** 10.3389/fneur.2023.1123603

**Published:** 2023-03-09

**Authors:** Ningxiang Qin, Xingguo Wu, Jing Wang, Wei Wang, Xuefeng Wang, Yuanlin Ma, Liang Wang

**Affiliations:** Department of Neurology, The First Affiliated Hospital of Chongqing Medical University, Chongqing, China

**Keywords:** autoimmune GFAP astrocytopathy, tuberculous meningitis, meningoencephalitis, anti-GFAP antibody, case report

## Abstract

**Introduction:**

Autoimmune glial fibrillary acidic protein (GFAP) astrocytopathy is a new form of autoimmunity-mediated central nervous system disease. It is especially easy to misdiagnose when clinical symptoms and cerebrospinal fluid (CSF) indicators are similar to those observed in patients with tuberculous meningitis (TBM).

**Methods:**

We retrospectively analyzed five cases of autoimmune GFAP astrocytopathy that were initially misdiagnosed as TBM.

**Results:**

In the five reported cases, all but one patient had meningoencephalitis in the clinic, and all patients exhibited increased pressure, lymphocytosis, increased protein levels, and decreased glucose levels in their CSF results and did not have typical imaging findings of autoimmune GFAP astrocytopathy. TBM was the initial diagnosis in all five patients. However, we found no direct evidence of tuberculosis infection, and anti-tuberculosis treatment had inconclusive effects. Following a GFAP antibody test, the diagnosis of autoimmune GFAP astrocytopathy was made.

**Conclusion:**

When there is a suspected diagnosis of TBM but TB-related tests are negative, the possibility of autoimmune GFAP astrocytopathy should be considered.

## 1. Introduction

Autoimmune glial fibrillary acidic protein (GFAP) astrocytopathy is a new type of autoimmune-mediated central nervous system disease that primarily affects the meninges, brain, spinal cord, and optic nerve. It is also associated with GFAP antibodies. It was first reported by Fang et al. in 2016 (1). The prevalence of autoimmune GFAP astrocytopathy is ~0.6 out of 100,000 individuals (2). In recorded cases, the patient age ranged from 8 to 103 years, with a median onset age between 44 and 54 years. The proportion of affected female individuals is nominally higher than that of male individuals (~55%), and children account for approximately 10% of patients (1, 3–5). Furthermore, no obvious racial differences exist (6). Although the etiology is unclear, autoimmune GFAP astrocytopathy can be associated with tumors or an infection, which is similar to other types of autoimmune encephalitis. Prodrome infection symptoms and signs, such as fever, runny nose, and sore throat, are present in ~ 40% of patients, and tumors may be present in 25% of patients (5, 6). In addition, the location and severity of the lesions are connected to the specific presentation of autoimmune GFAP astrocytopathy symptoms. Subacute onset meningitis, encephalitis, myelitis, or a combination of these syndromes are common clinical features of these patients (7). When patients with autoimmune GFAP astrocytopathy exhibit meningoencephalitis, the presentation is easily confused with that of infectious meningoencephalitis (8). When cerebrospinal fluid (CSF) results are similar to those in a patient with tuberculous meningitis (TBM), the diagnosis is easily confused with that of TBM. As a result, the disorder must be precisely identified in clinical settings (8). In this retrospective study, five cases of autoimmune GFAP astrocytopathy that were initially diagnosed as TBM were reviewed. This case series is a reminder to clinicians, particularly those in TBM-endemic regions, to be aware of the possibility of autoimmune GFAP astrocytopathy when a patient presents clinically with TBM but the test results do not support TBM.

## 2. Methods

Five adult patients with autoimmune GFAP astrocytopathy from the Department of Neurology at the First Affiliated Hospital of Chongqing Medical University from January 2020 to January 2022 were retrospectively analyzed. The study population satisfied the following criteria (1): the clinical manifestations were consistent with meningitis, encephalitis, myelitis, or a combination of the abovementioned syndromes, and tests for anti-GFAP antibodies were positive in the CSF and serum. The study was approved by the Ethics Committee of the First Affiliated Hospital of Chongqing Medical University, and either the patients themselves or their families signed informed consent forms.

The demographic characteristics, clinical manifestations, laboratory examinations, imaging examinations, treatment processes, prognoses, and other clinical data throughout the course of the disease were collected and analyzed as part of the study. Prognostic conditions were evaluated through telephone or outpatient follow-ups, and the modified Rankin Scale (mRS) was used to assess the outcome.

The presence of anti-GFAP antibodies, as well as the antibodies associated with autoimmune encephalitis, and antibodies against aquaporin 4 (AQP4) and myelin oligodendrocyte glycoprotein (MOG), was tested in the patient serum and CSF using an indirect immunofluorescence cell-based assay (CBA). Human embryonic kidney cells (HEK293) expressing antigens were used in a positive CBA. All antibody tests were carried out in our hospital's neurology laboratory.

Cerebrospinal fluid cytology was performed by staining with May Grunwald–Giemsa (MGG) and using the method of slide centrifugation; this modified Ziehl–Neelsen stain for CSF has been previously described as the Xijing Hospital method (9).

## 3. Results

### 3.1. Clinical features

The age of the five patients in this study ranged from 38 to 66 years, and there were four male patients (80%) and one female patient (20%). The five patients had fever before developing neurological symptoms, with the majority of the patients experiencing an acute or subacute onset. In addition, three patients complained of headaches. The patient in Case 2 experienced night sweats. The patient in Case 3 experienced exhaustion, and the patient in Case 4 experienced coughing and expectoration. The patients in both Cases 1 and 4 had reports of seizures and status epilepticus. Three patients suffered from mental symptoms that manifested as hallucinations and gibberish language. Each of the five patients had varying degrees of cognitive impairment and decreased consciousness. One patient (Case 1) required tracheal intubation ventilation to help with ventilation after developing respiratory failure. Another patient (Case 4) developed myelopathy during the course of the disease, which manifested as paraplegia and dysuria and was accompanied by paralytic intestinal obstruction. In all five patients, a stiff neck was present (Table 1).

**Table 1:** Clinical data of five patients.

**Clinical data**	**Case 1**	**Case 2**	**Case 3**	**Case 4**	**Case 5**
Sex	Male	Female	Male	Male	Male
Age (years)	51	66	65	38	51
Course of disease (days)	5	40	7	19	4
**Clinical symptoms**					
Fever	Yes	Yes	Yes	Yes	Yes
Headache	No	Yes	No	Yes	Yes
Seizures	Yes	No	No	Yes	No
Mental symptoms	No	Hallucinations and gibberish language	Hallucinations and gibberish language	No	Hallucinations and gibberish language
Cognitive impairment	Yes	Yes	Yes	Yes	Yes
Decreased consciousness	Yes	Yes	Yes	Yes	Yes
Weakness of limbs	No	Yes	No	Yes	No
Other symptoms	Hiccups, respiratory failure	Night sweats, dysphagia, urinary incontinence	Fatigue, dysuria	Fatigue, cough and expectoration, intestinal infarction, dysuria	Dysuria, constipation
Stiff neck	Yes	Yes	Yes	Yes	Yes
**Routine blood examinations**					
WBC (4–10 × 10^9^/L)	8.72	4.62	7.83	13.4	7.86
*N*% (40%−75%)	75.9	73	76↑	86.1↑	67.4
RBC (4.3–5.8 × 10^12^/L)	5.47	4.12	3.87	4.05	4.27
PLT (100–300 × 10^9^/L)	205	333	198	376	255
Serum sodium (135–145 mmol/L)	127	145	121	124	139
**CSF**					
Pressure (80–180 mmH_2_O)	340	198	380	330	250
Number of cells (0–10 × 10^6^/L)	29	178	90	238	122
Protein levels (0.12–0.6 g/L)	2.1	2.61	1.78	1.54	2.16
Glucose/blood glucose (mmol/L, >50%)	3.1/8.3	2.6/5.9	2.6/7.7	2.41/6.4	5.1/10.7
Chlorine (120–130 mmol/L)	109	120	106	113	117
Cytology	Lymphocytosis	Lymphocytosis	Lymphocytosis	Lymphocytosis, eosinophilia	Lymphocytosis
Modified Ziehl-Neelsen stain	Negative	Negative	Negative	Negative	Negative
**GFAP antibody**					
CSF	Positive	Positive	Positive	Positive	Positive
Serum	Positive	Positive	Positive	Positive	Positive
Other AE antibodies	Negative	Negative	Negative	Negative	Negative
Antinuclear antibody spectrum	Negative	Negative	Negative	Negative	Negative
X-Pert	Negative	ND	ND	ND	ND
T-SPOT	Positive	Positive	Negative	Negative	Negative
Cranial MRI	Abnormal	Abnormal	Abnormal	Abnormal	Abnormal
Tumor	No	No	No	No	No
Anti-tuberculosis time (days)	21	3	34	3	7
Anti-tuberculosis medicine	INH, RFP, PZA	INH, RFP, PZA	INH, RFP, PZA	INH, RFP, PZA	INH, RFP, PZA
Diagnosis time	23	7	34	3	7
**Treatment**					
Methylprednisolone	Yes	Yes	Yes	Yes	Yes
IVIg	Yes	No	No	Yes	Yes
Hospital stay (days)	25	25	36	53	41
**Prognosis**					
mRS score	Death	1	0	4	0

WBC, white blood cell; N%, neutrophil percentage; RBC, red blood cell; PLT, platelet; AE, autoimmune encephalitis; CSF, cerebrospinal fluid; GFAP, glial fibrillary acidic protein; mRS, modified Rankin Scale; ND, not detected; INH, isoniazid; PZA, pyrazinamide; RFP, rifampicin; IVIg, intravenous immunoglobulin.

Other AE antibodies included anti-NMDAR antibodies, anti-GABAB antibodies, anti-AMPAR antibodies, anti-LGI1 antibodies, and anti-CASPR2 antibodies.

### 3.2. Laboratory examinations

Routine blood examination: Only the patient in Case 4 exhibited elevated white blood cell counts during the early stages of the disease.

#### 3.2.1. CSF analysis

All five patients experienced an increase in CSF pressure (range: 200–380 mmH_2_O, reference range 80–180 mmH_2_O), an increase in the number of nucleated cells (range: 29–238 × 106/L, reference range: 0–10 × 10^6^/L), an increase in protein levels (range: 1.54–2.61 g/L, reference range: 0.12–0.6 g/L), a decrease in chloride levels (range: 106–120 mmol/L, reference range: 120–130 mmol/L), and a decrease in glucose levels (range: 34%−48% of blood glucose, reference range: >50%). Both CSF culture and routine bacterial smear results were negative. Furthermore, CSF cytology revealed an inflammatory response with lymphocytosis. The modified acid-fast staining results for CSF were negative. Furthermore, two of the five patients had positive results for the T-SPOT (Case 1 and Case 2). Three patients (Cases 1, 3, and 4) developed moderate hyponatremia, with the patient in Case 3 having a diagnosis of syndrome of inappropriate antidiuretic hormone (SIADH) and requiring admission to the endocrinology department.

#### 3.2.2. Antibody test

All five patients tested positive for GFAP antibodies but negative for other antibodies associated with autoimmune encephalitis (both in the CSF and serum). The patient in Case 4 underwent MOG and AQP4 antibody tests using serum and CSF due to myelitis symptoms, but the results were negative. Furthermore, all of the patients tested negative for serum tumor markers as well as the antinuclear antibody spectrum (Table 1).

### 3.3. Imaging

**Figure 1:**
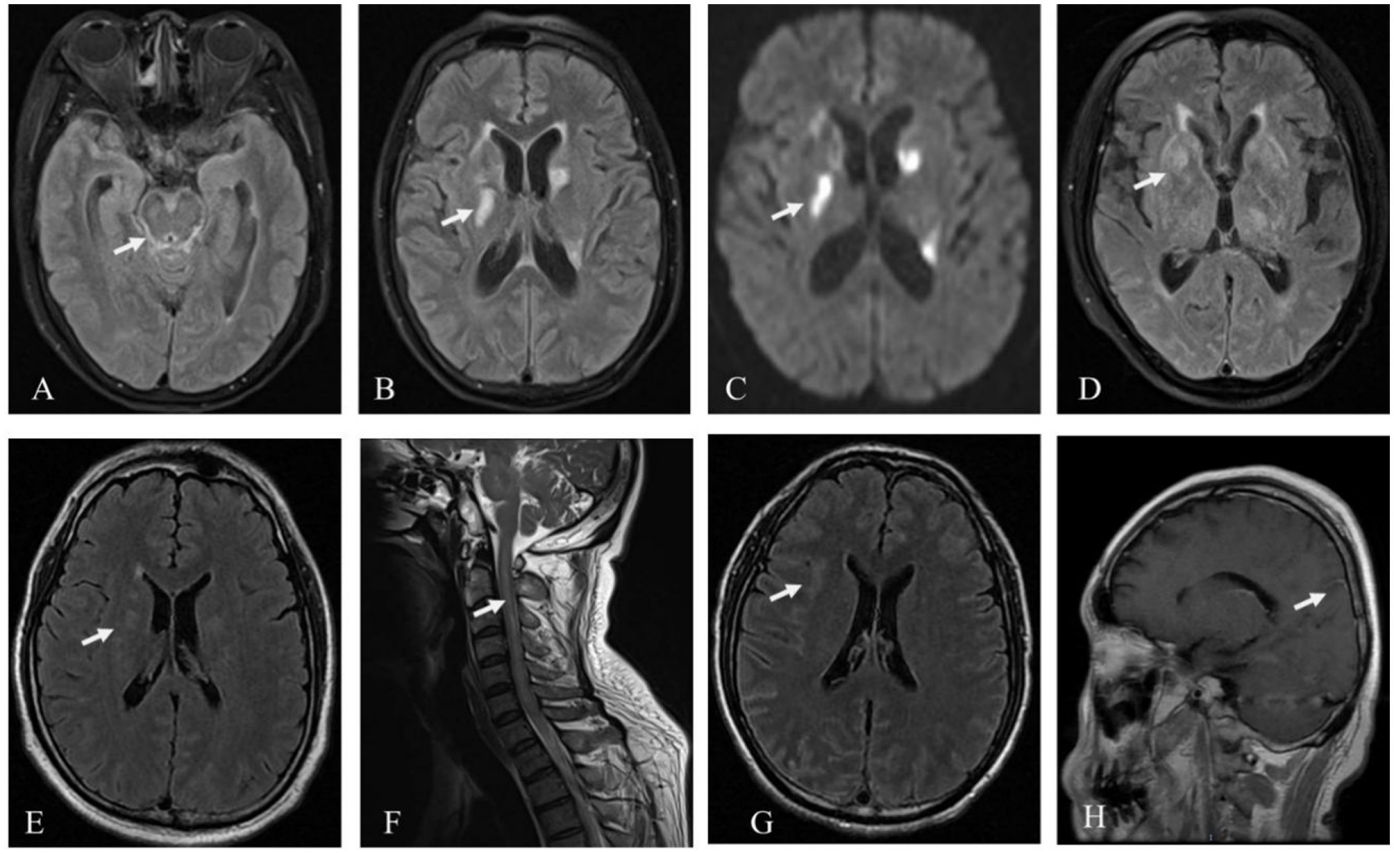
Patient MRI images. Hyperintensity signal inthe brainstem, pia mater, and basal ganglia on T2 FLAIR **(A, B)** and the basal ganglia on DWI in the patient in Case 2 **(C)**; hyperintensity signal in the basal ganglia on T2 FLAIR in Case 3 **(D)**; hyperintensity signal in the periventricular white matter on T2 FLAIR **(E)**, and the cervical spinal cord on T2 in the patient in Case 4 **(F)**; hyperintensity signal in the subcortex on T2 FLAIR and parieto-occipital sulci meningeal enhancement on T1 in the patient in Case 5 **(G, H)**.

Except for the patient involved in Case 1 (who had a history of brain trauma surgery and was suggested to have a focus on encephalomalacia), the other four patients exhibited various abnormal signals, mostly involving the subcortex, periventricular white matter, basal ganglia, and brainstem, among other areas on cranial MRI (Figure 1). Furthermore, four patients' MRI results showed a high signal or equal signal on T1-weighted images and a high signal on T2-weighted and T2 FLAIR images. The patient in Case 2 exhibited a bright signal on diffusion-weighted imaging (DWI) and a black signal on ADC in the basal ganglia and lateral ventricle, indicating cytotoxic edema in these lesions. Moreover, in the patients in Cases 2, 4, and 5, high T2 FLAIR signals were observed in the sulcus and pia mater. The cervical spinal cord MRI of the patient in Case 4 exhibited a high signal on T2-weighted images. Gadolinium-enhanced brain MRI was performed on all five patients, and the patient in Case 5 exhibited parieto-occipital sulci meningeal enhancement. There was no linear perivascular radial enhancement pattern in any of the patients. Chest and abdomen CT scans, as well as urinary color Doppler ultrasound, were used to screen each patient for tumors, but no space-occupying lesions were found.

### 3.4. Misdiagnosis process, treatment, and outcome

The five patients were initially diagnosed with TBM and were given anti-tuberculosis medication. The duration of anti-tuberculosis treatment ranged from 3 to 34 days. Specifically, the anti-tuberculosis therapy regimen included isoniazid (INH) at 1,000 mg intravenous drip, rifampicin (RIF) at 450 mg oral, pyrazinamide (PZA) at 1,500 mg oral, and dexamethasone (DXM) at 10 mg intravenous drip for anti-inflammatory treatment. Except for the patient in Case 1, who demonstrated a lung infection that worsened after anti-tuberculosis medication and who had symptoms that did not significantly improve, the symptoms of the other four patients did not worsen; instead, they slightly improved. After the anti-GFAP antibodies in the serum and CSF were detected, all patients were diagnosed with autoimmune GFAP astrocytopathy between 3 and 24 days after their initial diagnosis. The patients were given methylprednisolone at 1,000 mg daily for 3 days and intravenous immunoglobulin (IVIg) at 0.4 g/(kg·d) for 5 days. The length of stay in the hospital ranged from 25 to 53 days. Unfortunately, the patient in Case 1 died due to respiratory failure. The patient in Case 5 received additional therapy at a rehabilitation hospital after being discharged with grade 2 muscle strength in the lower limbs. The other three patients were discharged with mRS scores of 0 or 1 (Table 1).

## 4. Discussion

Prodrome symptoms of autoimmune GFAP astrocytopathy, such as fever and headache, are present in 40% of patients, and meningoencephalitis is the predominant clinical condition in 55% of cases (5, 10). The diagnostic criteria for autoimmune GFAP astrocytopathy include meningitis, encephalitis, or myelitis (or a combination thereof) and anti-GFAP antibody positivity in CSF (7). The five cases of patients presented in this report had meningoencephalitis and tested positive for anti-GFAP antibodies in both their serum and CSF. As a result, they were ultimately diagnosed with autoimmune GFAP astrocytopathy.

If patients have clinical symptoms of meningitis or meningoencephalitis, autoimmune GFAP astrocytopathy must be distinguished from infectious meningitis or meningoencephalitis. CSF findings of leukocytosis (>50 × 10^6^/L), increased protein (>1 g/L), and hypoglycemia (<50% blood glucose during the testing period) are frequently indicative of tuberculous meningoencephalitis. In addition to the five cases reported here, other researchers have reported cases of autoimmune GFAP astrocytopathy that were initially misdiagnosed as infectious meningitis or tuberculous meningoencephalitis (11–13). Because autoimmune GFAP astrocytopathy and TBM have extremely similar clinical manifestations and features, it is critical to focus on identifying antibodies in the CSF and direct signs of tuberculosis infection to differentiate these conditions. Although detecting anti-GFAP antibodies in the CSF or serum is the primary method for diagnosing autoimmune GFAP astrocytopathy, antibody detection has not been widely adopted in clinical settings due to its high cost and the restrictions imposed by medical insurance. In addition, diagnosis of TBM requires an etiological diagnosis, but it is less likely to find *Mycobacterium tuberculosis* in the CSF or to identify *M. tuberculosis* DNA *via* PCR.

The five patients in our study were given a likely diagnosis of TBM based on the Vietnam TBM diagnostic scoring system from 2010 (14). Notably, when patients with autoimmune GFAP astrocytopathy have low CSF glucose levels, they are frequently suspected of having infectious encephalitis. A decreased glucose level was reported in the CSF of nine of the 59 patients with autoimmune GFAP astrocytopathy (15.25%) (8, 15–19). The precise mechanism and clinical importance of hypoglycorrhachia in autoimmune GFAP astrocytopathy remain unknown. Furthermore, lymphocytosis and an absence of lymphoma cells were found in the CSF of these five patients. Except for an increase in neutrophil levels during the very early stages of TBM, cytology primarily shows lymphocyte reactions throughout the course of the disease. Although some researchers have shown that autoimmune GFAP astrocytopathy involves a specific proportion of eosinophils in the CSF (8), eosinophils are nonspecific as a differential indicator because many factors, such as allergies, parasites, or tumors, can cause increased levels of these cells. In addition, T-SPOT is an ELISA test that uses tubercle bacillus (TB)-specific antigen to identify T lymphocytes in the peripheral blood that are specific to the TB antigen. Although T-SPOT has high sensitivity, it cannot be used to differentiate between ongoing infections, prior infections, or latent infections and therefore cannot yield a precise or unique diagnosis of tuberculosis (20). The T-SPOT-positive results in Cases 1 and 2 did not indicate an active TB infection. Patients with TBM are more likely to have hyponatremia, especially SIADH (21). Three of the five patients had moderate hyponatremia, with the patient in Case 3 being diagnosed with SIADH and the plasma sodium levels returning to normal after water restriction. In autoimmune encephalitis, anti-LGI1 antibody encephalitis is frequently associated with SIADH (22). Previous studies have reported hyponatremia in patients with autoimmune GFAP astrocytopathy (3, 5). More cases need to be analyzed to determine whether hyponatremia is a common symptom of autoimmune GFAP astrocytopathy.

The majority of patients with autoimmune GFAP astrocytopathy have abnormalities that can be identified using cranial MRIs, and the lesions can affect several brain regions, including the cerebellum, basal ganglia, hypothalamus, periventricular white matter, and meninges (23). The most specific imaging manifestation of autoimmune GFAP astrocytopathy is linear radial perivascular enhancement surrounding the lateral ventricle found in approximately half of the patients during enhanced MRI of the head (5). TBM imaging manifestations include hydrocephalus, meningeal enhancement, tuberculoma, infarcts, and basal ganglia calcification (24). In the five cases reported in this study, the longitudinal radial perivascular enhancement that typically characterizes autoimmune GFAP astrocytopathy surrounding the lateral ventricle was not present. In Case 2, DWI demonstrated restricted diffusion in the basal ganglia and lateral ventricle, which is uncommon for autoimmune GFAP astrocytopathy and more suggestive of TBM. Therefore, in the absence of measurable imaging changes, it is difficult to distinguish autoimmune GFAP astrocytopathy from TBM.

## 5. Conclusion

Five adult patients with autoimmune GFAP astrocytopathy misdiagnosed as TBM are summarized herein. Based on these cases as well as previous research, a certain percentage of autoimmune GFAP astrocytopathy and TBM manifestations are likely to be confused, especially when there is no direct evidence of relevant antibodies or TB infection. In regions where TBM is endemic, it is necessary to consider the possibility of autoimmune GFAP astrocytopathy when a patient's clinical symptoms and routine biochemical examinations in the CSF are similar to those of TBM but other examinations related to tuberculosis are negative and there are nonspecific changes in imaging.

## Data availability statement

The original contributions presented in the study are included in the article/supplementary material, further inquiries can be directed to the corresponding author.

## Ethics statement

The studies involving human participants were reviewed and approved by the Ethics Committee of the First Affiliated Hospital of Chongqing Medical University. The patients/participants provided their written informed consent to participate in this study. Written informed consent was obtained from the individual(s) for the publication of any potentially identifiable images or data included in this article.

## Author contributions

NXQ: paper writing and data collection. XGW: paper writing and statistics. JW and YLM: data collection. WW: statistics. XFW: project design. LW: project design and paper writing. All authors contributed to the article and approved the submitted version.
